# A novel *ANO3* variant identified in a 53-year-old woman presenting with hyperkinetic dysarthria, blepharospasm, hyperkinesias, and complex motor tics

**DOI:** 10.1186/s12881-016-0354-7

**Published:** 2016-12-05

**Authors:** Patrick R. Blackburn, Michael T. Zimmermann, Jennifer M. Gass, Kimberly G. Harris, Margot A. Cousin, Nicole J. Boczek, Owen A. Ross, Eric W. Klee, Paul W. Brazis, Jay A. Van Gerpen, Paldeep S. Atwal

**Affiliations:** 1 Center for Individualized Medicine, Mayo Clinic, Jacksonville, FL USA; 2 Department of Health Sciences Research, Mayo Clinic, Jacksonville, FL USA; 3 Department of Health Sciences Research, Mayo Clinic, Rochester, MN USA; 4 Department of Neuroscience, Mayo Clinic, Jacksonville, FL USA; 5 Center for Individualized Medicine, Mayo Clinic, Rochester, MN USA; 6 Department of Clinical Genomics, Mayo Clinic, 4500 San Pablo Road South, Jacksonville, FL 32224 USA; 7 Department of Clinical Genomics, Mayo Clinic, Rochester, MN USA; 8 Department of Ophthalmology, Mayo Clinic, Jacksonville, FL USA; 9 Department of Neurology, Mayo Clinic, Jacksonville, FL USA

**Keywords:** ANO3, Anoctamin-3, Dystonia-24, DYT24, Craniocervical dystonia

## Abstract

**Background:**

Cervical dystonias have a variable presentation and underlying etiology, but collectively represent the most common form of focal dystonia. There are a number of known genetic forms of dystonia (DYT1-27); however the heterogeneity of disease presentation does not always make it easy to categorize the disease by phenotype-genotype comparison.

**Case presentation:**

In this report, we describe a 53-year-old female who presented initially with hand tremor following a total hip arthroplasty. The patient developed a mixed hyperkinetic disorder consisting of chorea, dystonia affecting the upper extremities, dysarthria, and blepharospasm. Whole exome sequencing of the patient revealed a novel heterozygous missense variant (Chr11(GRCh38): g.26525644C > G; NM_031418.2(ANO3): c.702C > G; NP_113606.2. p.C234W) in exon 7 in the *ANO3* gene.

**Conclusions:**

*ANO3* encodes anoctamin-3, a Ca^+2^-dependent phospholipid scramblase expressed in striatal-neurons, that has been implicated in autosomal dominant craniocervical dystonia (Dystonia-24, DYT24, MIM# 615034). To date, only a handful of cases of DYT-24 have been described in the literature. The complex clinical presentation of the patient described includes hyperkinesias, complex motor movements, and vocal tics, which have not been reported in other patients with DYT24. This report highlights the utility of using clinical whole exome sequencing in patients with complex neurological phenotypes that would not normally fit a classical presentation of a defined genetic disease.

**Electronic supplementary material:**

The online version of this article (doi:10.1186/s12881-016-0354-7) contains supplementary material, which is available to authorized users.

## Background

Dystonias are a heterogeneous group of movement disorders with both primary genetic and secondary environmental etiologies [1]. Over the last few decades, several novel disease associated genes (DYT1-27) have been identified in dystonic syndromes, but the underlying genetic diagnosis remains elusive in most patients [1]. Inherited isolated craniocervical dystonias are rare, and most commonly caused by pathogenic variants in *THAP1* (Dystonia-6, DYT6, MIM# 602629) and *GNAL* (Dystonia-25, DYT25, MIM# 615073) and have adolescent to late adult onset with variable penetrance [2]. To date, targeted clinical gene testing has been performed with limited success, however with the advent of next generation sequencing technologies in the clinic, we are beginning to unravel the complex genetic landscape of primary dystonias.

Using exome sequencing, Charlesworth et al. [3] identified pathogenic variants in the anoctamin-3 gene (*ANO3*) in three families in the UK with craniocervical dystonia, including the index family described in Münchau et al. [3, 4]. The age at onset ranges from early childhood to the 5th decade with patients typically presenting in the late 4th decade of life with cervical and laryngeal dystonia (Table 1) [5]. Most affected individuals also have dystonic tremor that affects the upper limbs, which can be misdiagnosed as familial essential tremor [5]. Patients can also develop ataxia, head tremor, dystonic posturing of the upper limbs, oromandibular dystonia, dysarthria, blepharospasm, and mild cognitive impairment. Interestingly, in at least one family, an unaffected *ANO3* pathogenic variant carrier had both an affected child and an affected parent, suggesting reduced penetrance [5].

**Table 1:**
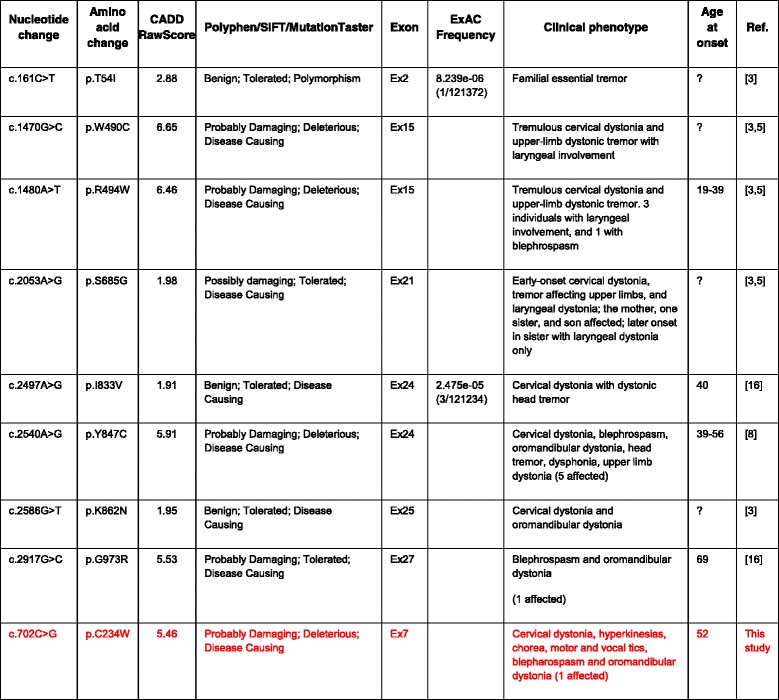
Previously described *ANO3* variants that are likely associated with disease

The novel variant described in this report is highlighted in red

**Fig. 1:**
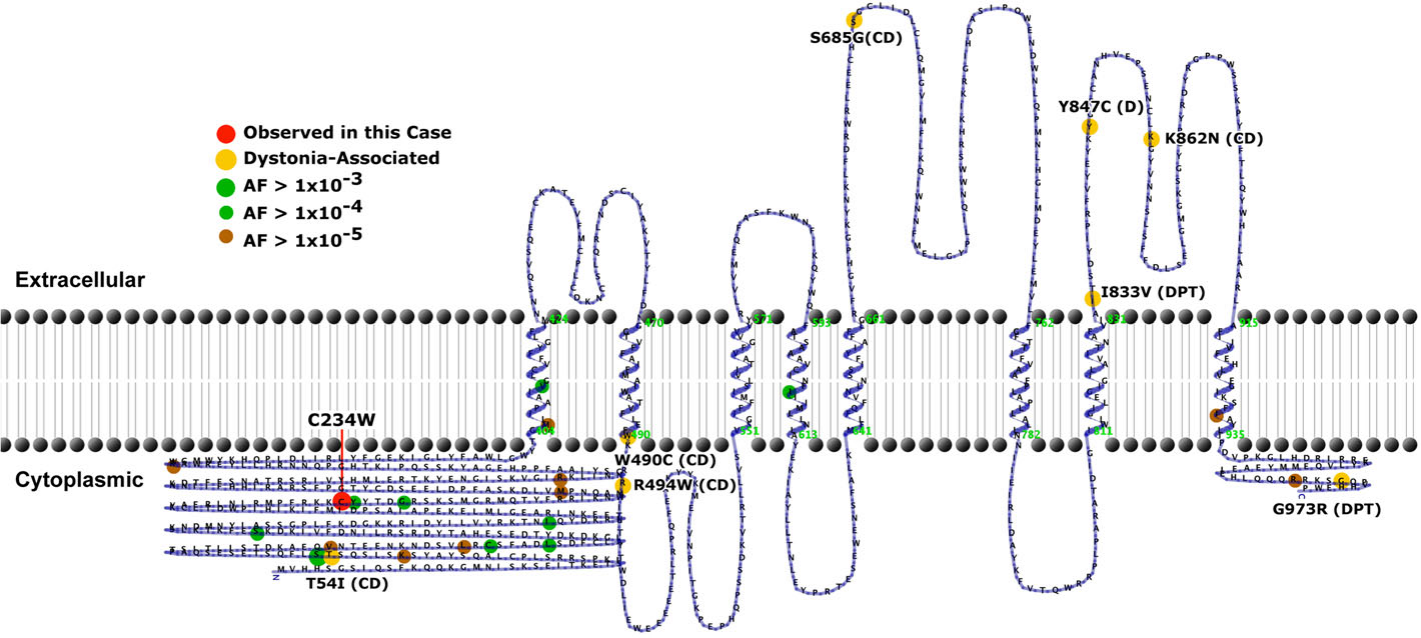
Summary of previously reported ANO3 variants. We used TMRPres2D to generate a schematic layout of ANO3 and color to annotate the protein sequence using ExAC allele frequencies and the eight previously reported dystonia-associated missense mutations [19]. The HGMD database associates these variants with dystonia (D), craniocervical dystonia (CD), or dystonia primary torsion (DPT) [20]

*ANO3* encodes anoctamin-3, a homodimeric protein belonging to the anoctamin/TMEM16 family of proteins that are structurally related and encode Ca^+2^-activated chloride channels and membrane phospholipid scramblases with distinct patterns of expression [6]. ANO3 consists of eight hydrophobic transmembrane helices and may act as a Ca^+2^ sensor involved in regulating calcium homeostasis (Fig. 1) [6]. The exact function of ANO3 is still poorly understood, and recent experiments have shown that it does not act as a Ca^+2^-activated chloride channel, and may in fact function as a Ca^+2^-dependent phospholipid scramblase [7]. ANO3 appears to have a role in the modulation of neuronal excitability and is highly expressed in the striatum, hippocampus, and cortex. [3, 6] Mechanistically, pathogenic variants in *ANO3* could lead to abnormal striatal-neuron excitability, which manifests as uncontrolled dystonic movements [6].

In this report we describe a patient with atypical craniocervical dystonia presenting with chorea and complex motor tics with a novel variant (Chr11(GRCh38): g.26525644C > G; NM_031418.2(ANO3): c.702C > G; NP_113606.2. p.C234W) in *ANO3* that was identified using whole exome sequencing (WES).

## Case presentation

The patient is a 53-year-old white female who presented at 52-years-of-age with tremor following a right total hip arthroplasty. Postoperatively, the patient had nausea from her pain medication and was on promethazine for several months when she first noted her hand tremor. Promethazine exposure could be associated with the genesis of her hyperkinesias; however, her tremor quickly progressed to diffuse abnormal choreiform movements affecting her upper extremities and torso with increasing frequency (Additional file 1: Video S1). Concurrently, the patient also noted difficulty with speech and extreme sensitivity to light, which preceded the development of blepharospasm. The patient had difficulty focusing without any noticeable decrease in visual acuity. She described difficulty focusing on visual stimuli in open spaces with accentuation of her diffuse abnormal body movements.Additional file 1: Video S1. Patient history and tremor. Video of patient tremor with diffuse abnormal choreiform movements affecting her upper extremities and torso. (WMV 19181 kb)


Upon examination, the patient had a tendency toward phasic, left torticollis and had developed hypertrophy and tightness of the strap muscles that was becoming painful. The patient had mixed, generalized hyperkinesias and cervical dystonia. She also had evidence of abnormal posturing within her phalanges and left hand, which were suggestive of multi-focal dystonia. She performed movements, such as clapping her hands, which were thought to reflect complex motor tics (Additional file 2: Video S2). Her gait was slow and she took small steps during the exam. The patient frequently grimaced and reported that she “cracked several teeth,” both secondary to her orofacial dystonia. Motor speech examination provoked accelerated, uncontrolled upper extremity movements. With tongue protrusion, there was evidence of motor impersistence (Additional file 3: Video S3). The patient also showed slowed processing speed, dissociation of knowledge from action, and echopraxia that were thought to be consistent with frontal lobe involvement. At the time, the patient was taking clonazepam, carbidopa/levodopa, trazodone, naproxen, and hydrochlorothiazide with some benefit.Additional file 2: Video S2. Patient gait and complex motor tics. Video of patient performing movements while walking, such as clapping her hands, which reflect complex motor tics. The patient’s lower extremities were unaffected. (M4V 3657 kb)
Additional file 3: Video S3. Motor impersistence with tongue protrusion. With tongue protrusion. (WMV 15275 kb)


Initial workup of the patient for secondary causes of dystonia was unrevealing and included complete blood count (CBC) with peripheral smear (no acanthocytes seen), normal sedimentation rate, vitamin B12, methylmalonic acid, electrolytes, ammonia, ceruloplasmin, copper, liver function tests, thyroid-stimulating hormone (TSH), paraneoplastic profile, tests of connective tissue disorders, sera rapid plasma reagin (RPR), negative anti-thyroid peroxidase (TPO) antibodies and tissue transglutaminase antibodies. Additionally, the patient’s local physician reported genetic testing for Huntington’s disease that was normal.

### Family history

**Fig. 2:**
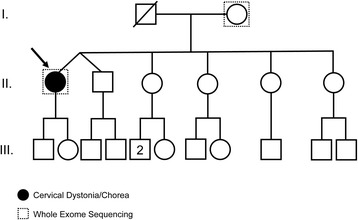
Three-generation family pedigree showing the proband and relatives. Both the proband (*arrow*) and her 85-year-old mother had whole exome and mitochondrial DNA sequencing and the clinically reportable results are shown in Table 2

The patient’s father was deceased and therefore unavailable for testing. The family history is otherwise unremarkable (Fig. 2). We were unable to get additional family members to participate in the research study, including the patient’s siblings. Of note, both sporadic and familial cases of *ANO3*-related cervical dystonia have been observed in the literature highlighting the reduced penetrance associated with this form of dystonia [3, 8].

### Ophthalmological findings

The patient had no history of diplopia, oscillopsia, or history of previous ophthalmologic disease processes. The patient’s neuro-ophthalmologic examination revealed normal visual acuity and color vision. The patient was noted to have constant bilateral forcible eyelid closure and it was difficult to demonstrate any definite apraxia of eyelid opening or eye closure. The patient was noted to have bilateral upward deviation of the eyes that was consistent with physiologic Bell’s phenomenon. The patient’s ocular motility was full with lateral gaze intact. Occasionally she developed an esotropia with constriction of the pupils compatible with spasm of the near reflex. The patient did not have nystagmus. Cranial nerves V and VII were intact except for occasional abnormal facial movements and frequent eye closure.

### Speech/language assessment

The patient’s voice was hypophonic, with high-pitch and strained stuttering speech (Additional file 1: Video S1). She repeated consonants at the beginning of some sentences and had some elongated vowel sounds as well. The patient understood what was said to her and despite her challenges with speech, she was able to communicate her ideas although she exhibited echopraxia. She also had several episodes of spontaneous crying that were suggestive of a pseudobulbar component to her disease. The patient exhibited perceptual evidence of a moderate dysarthria, with clinical features that appear compatible with a hyperkinetic dysarthria. The patient’s receptive and expressive language skills were unimpaired, but her writing legibility and reading from computer screen were affected due to uncontrolled upper extremity movements, as well as visual sensitivity.

### Electrophysiology

The EEG was moderately abnormal due to the presence of excessive myogenic activity. The patient was tense and experienced a number of abnormal movements (tremor, jerks) that were not associated with epileptiform activity. These were only associated with movement and myogenic artifacts but baseline activity was maintained. The background activity was predominantly around 7 Hz and was intermixed with beta activity that was symmetrical and reactive. There was excessive beta activity that was thought to be due to medication effect as the patient was taking benzodiazepine medication. The background activity was mainly in theta frequency band and was thought to represent either medication effect or a more organic pathology such as encephalopathy.

### Genetic testing

Clinical WES was performed by GeneDX (XomeDxPlus), which also included mitochondrial DNA sequencing. Briefly, genomic DNA was extracted from blood from the proband and her mother. As described in the clinical testing methodology by GeneDX, the SureSelectXT Clinical Research Exome (Agilent) capture kit was used for exome enrichment and sequencing was done on an Illumina HiSeq 2000 that generates 100 bp paired-end reads. Bi-directional sequence was assembled, aligned to reference gene sequences based on human genome build GRCh37/UCSC hg19, and analyzed for sequence variants using a proprietary analysis tool (Xome Analyzer, GeneDx) as previously described [9]. Sanger sequencing was used to confirm all potentially pathogenic variants identified in this individual and in the parental sample [9]. Sequence alterations were reported according to the Human Genome Variation Society (HGVS) nomenclature guidelines. The exome was covered to a mean depth of 97×, with a quality threshold of 95.7%.

The patient was found to have a c.702C > G, p.C234W missense variant in exon 7 (of 27 total exons) in the *ANO3* gene that falls within the cytoplasmic N-terminus (Fig. 1). This variant was not found in the patient’s mother and testing was not performed on the patient’s father who is deceased. For this gene, 100% of the coding region was covered at a minimum of 10× by the XomeDx test (GeneDX). The c.702C > G variant in the *ANO3* gene has not been observed in approximately 6500 individuals of European and African American ancestry in the NHLBI Exome Sequencing Project or in over 60,000 individuals in ExAC [10, 11]. No other variants were reported in the clinical sequencing report and mitochondrial DNA sequencing revealed a m.8999 T > C p.V158A variant of uncertain significance in *MT-ATP6* that was homoplasmic in both the proband and her 85-year-old mother (Table 2).

**Table 2:** Clinically reportable variants found within the patient by whole exome sequencing or mitochondrial DNA sequencing

Gene	NCBI accession number	Nucleotide change	Amino acid change	Exon	Inheritance	Zygosity	ACMG classification
ANO3	NM_031418.2	c.702C > G	p.C234W	Ex7	Unknown	Heterozygous	VUS
MT-ATP6	NC_012920.1	c.473 T > C	p.V158A	Ex1	Maternal	Homoplasmic	VUS

**Fig. 3:**
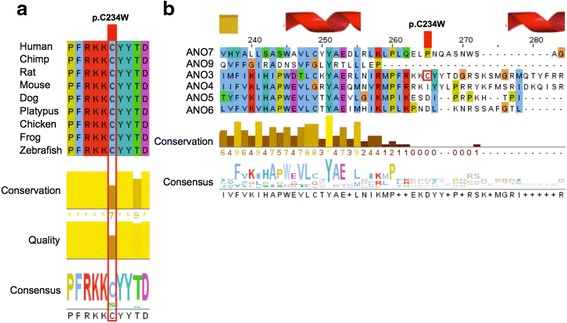
Annotated multiple sequence alignments (MSA) for ANO3 homologs and human ANO3 paralogs. **a** ANO3 homologs show a high degree of conservation across species (only select species shown; coloring by amino acid type). The site is either C or S from human, through hominids, rodents, and whales (shown by conservation and consensus logo) across 69 different species (identified by pBLAST [21] and aligned using COBALT [22]; data not shown). **b** Human ANO3 paralogs show some level of conservation in the region preceding the variant of interest (p.C234W; shown as a *red box*), but the site itself is not conserved across human paralogs. Both figures were created using Jalview [23]

The p.C234 residue in ANO3 is moderately conserved across species and falls within a region that is highly conserved among homologs (Fig. 3a), the only variants at position 234 being a conservative C > S present in some ungulate and whale species, but is not well represented across human ANO3 paralogs (Fig. 3b). The *in silico* prediction algorithms, SIFT, PolyPhen-2, and MutationTaster2 predict this missense mutation to be deleterious, possibly damaging, and disease causing, respectively (Table 1) [12–14]. The Combined Annotation Dependent Depletion (CADD v1.3) raw score for this variant is 5.46 and ranges from 1.91 to 6.65 for the other previously reported variants in *ANO3* (scores range from 1 to 99, with a higher score indicating a greater likelihood of being deleterious) (Table 1) [15]. In ExAC, the probability of being loss-of-function (LoF) intolerant value (pLI) for ANO3 is 0. The pLI provides a measure of a given gene’s intolerance to variation and controls for coding sequence length (pLI ≥ 0.9 may indicate LoF intolerant genes and pLI ≤ 0.1 may indicate LoF tolerant ones) [11]. This metric may provide an indication of whether heterozygous LoF variants would be expected confer some survival or reproductive disadvantage but does not necessarily reflect the ability of a gene to result in disease (for instance the prion protein gene, *PRNP*, which is associated with a number of autosomal dominant neurodegenerative spongiform encephalopathies has a pLI of 0.03 and is predicted to be relatively tolerant of coding variation) [11]. In the patient’s WES report the p.C234W missense variant is classified as a variant of uncertain significance. Given the strong clinical and phenotypic overlap with previously published DYT24 patients, however, this variant is a strong candidate in the etiology of this patient’s disease. In order to rule out other genetic causes of disease, we looked at the coverage of other genes that have been associated with cervical dystonia. *GNAL* (Dystonia-25, DYT25, MIM# 615073), *THAP1* (torsion dystonia-6, DYT6, MIM# 602629), *TOR1A* (torsion dystonia-1, DYT1, MIM# 128100), *CIZ1*, *HPCA* (torsion dystonia-2, DYT2, MIM# 224500), *TUBB4A* (torsion dystonia-4, DYT4, MIM# 128101) and *COL6A3* (dystonia-27, DYT27, MIM# 616411) had 100% coverage by WES and no reportable variants were identified in any of these genes. However, WES could not rule out deletions or duplications affecting these genes.

## Conclusions

In this report we describe a 53-year-old female patient with a novel heterozygous missense variant of uncertain significance (VUS) (Chr11(GRCh38): g.26525644C > G; NM_031418.2(ANO3): c.702C > G; NP_113606.2. p.C234W) in *ANO3* who had a late and precipitous onset of disease. The patient shares many of the same clinical and pathological features as patients described with autosomal dominant craniocervical dystonia, including initial manifestation in the form of a progressive tremor, with development of a dystonia affecting the upper extremities, dysarthria, and blepharospasm. However, the patient also has mixed hyperkinesias manifesting as chorea, as well as simple and complex motor and vocal tics, which have not been observed in other patients with DYT24. Potentially complicating the patient’s phenotype is the fact that she was on promethazine for several months following a total right hip arthroplasty. Her hyperkinesias became evident while she was on the promethazine thus, leading to speculation that some of her hyperkinesias could have a tardive etiology.

The p.C234W *ANO3* variant described in this patient is classified as a VUS by clinical report, is predicted to be damaging by *in silico* analysis, and has never been reported in any publically available databases. Given the good phenotypic overlap, we posit that this variant may contribute to the patient’s disease. However, a recent study identified a neighboring c.704A > G (p.Y235C) missense mutation in 4 of 4300 European American individuals within the NHLBI-ESP cohort [16]; the variant is also reported 35 times (out of 114122 alleles) in ExAC database. As yet there is no report of the p.Y235C variant linked with disease, even though it is rare and predicted to be damaging [16]. It remains possible that with the noted reduced penetrance and later onset of tremor observed in some families, that the more benign manifestations of DYT24 could go undetected in a seemingly healthy control population.

To date, 8 pathogenic missense variants in *ANO3* have been identified including: c.2540A > G (p.Y847C), c.1480A > T (p.R494W), c.1470G > C (p.W490C), c.161C > T (p.T54I), c.2053A > G (p. S685G), c.2586G > T (p.K862N), c.2190C > T, c.2497A > G (p.I833V), c.2917G > C (p.G973R) (Table 1) [8]. These variants fall within the transmembrane spanning alpha helices, within the intracellular and extracellular loops, and within the N and C-termini of ANO3 (Fig. 1). While these variants do not implicate a hotspot, their spatial relationships within the 3D protein structure are unknown (Fig. 1). Future work investigating the 3D structure could shed light on common mechanisms of alteration by each variant. No nonsense or frame-shift mutations in *ANO3* have been reported in association with DYT24, however there are several rare frameshift and nonsense variants present in ExAC, suggesting that there may be additional phenotypes associated with this gene such as autism spectrum disorders [17].

The ANO3 p.C234W substitution in the patient under study is located within the N-terminus of the protein (amino acids #1-403). The only other variant described in the N-terminus (NM_031418.2, exon 2, c.161C > T, p.T54I) was in a patient who was diagnosed with familial essential tremor (Table 1) [3]. The function of the N-terminal region of ANO3 and other anoctamin family members remains poorly described, but may be involved in dimerization or interactions with other proteins such as calmodulin, as has been demonstrated in ANO1 (TMEM16A) [6].

Only the c.1470G > C (p.W490C) variant has been evaluated using functional studies, with patient fibroblasts showing reduced ATP- and thapsigargin-induced calcium signal compared to controls, that was thought to be due to a smaller calcium pool in the endoplasmic reticulum [3]. ANO3 is expressed throughout the central and peripheral nervous system. In one study, rats were shown to have high Ano3 expression in a subset of nociceptive neurons in dorsal root ganglia (DRG) [6, 18]. Ano3 knockout rats (*Ano3* ^*−/−*^) were hypersensitive to high temperatures and electrophysiological measurement from DRG neurons from these animals showed action potential broadening and lower threshold for action potential firing [6, 18]. Interestingly, Na^+^-activated K^+^ current was also strongly reduced in *Ano3* ^*−/−*^ rats [6, 18]. Colocalization experiments showed that Ano3 directly interacts with Kcnt1 (Slack), a sodium-activated potassium channel implicated in infantile epileptic encephalopathy-14 (EIEE14, MIM# 614959) [6, 18]. Ano3 may enhance the activity of Kcnt1, which in turn helps regulate the excitability of nociceptive neurons [6, 18].

As we see an increase in the utilization of whole-exome and -genome sequencing in the clinic, there will be an ever-increasing demand for methods of determining disease relevance and pathogenicity. In this case report we identified a novel mutation of likely pathogenicity in a gene known to present with a similar phenotype. For rare protein variants such as ANO3 p.C234W, clinical genetic studies may not be sufficient to prove pathogenicity, rather additional functional studies will likely be needed. However with this in mind, it is critical that robust functional assays are developed that truly reflect the underlying disease mechanisms occurring, that is to say not all functional effects are created equally. As we gain a better understanding of the pathways and mechanisms underlying DYT24, and dystonia in general, clarification of rare variants will better direct targeted drug design and clinical trials.

## Abbreviations

ANO3: Anoctamin-3
CBC: Complete blood count
DRG: Dorsal root ganglia
DYT24: Dystonia-24
LoF: Loss-of-function
pLI: Probability of loss-of-function intolerant value
RPR: Rapid plasma regain
TPO: Anti-thyroid peroxidase
TSH: Thyroid-stimulating hormone
VUS: Variant of uncertain significance
WES: Whole exome sequencing
